# *Hunter disease eClinic: *interactive, computer-assisted, problem-based approach to independent learning about a rare genetic disease

**DOI:** 10.1186/1472-6920-10-72

**Published:** 2010-10-25

**Authors:** Fatma Al-Jasmi, Laura Moldovan, Joe TR Clarke

**Affiliations:** 1 Department of Pediatric, Faculty of Medicine and health Science, United Arab Emirates University, United Arab Emirates; 2 Division of Clinical and Metabolic Genetics, Toronto, Ontario, Canada; 3 Research Institute, Hospital for Sick Children, Toronto, Ontario, Canada; 4 Institute of Medical Sciences and Department of Pediatrics, University of Toronto, Toronto, Ontario, Canada

## Abstract

**Background:**

Computer-based teaching (CBT) is a well-known educational device, but it has never been applied systematically to the teaching of a complex, rare, genetic disease, such as Hunter disease (MPS II).

**Aim:**

To develop interactive teaching software functioning as a virtual clinic for the management of MPS II.

**Implementation and Results:**

The *Hunter disease eClinic*, a self-training, user-friendly educational software program, available at the Lysosomal Storage Research Group (http://www.lysosomalstorageresearch.ca), was developed using the Adobe Flash multimedia platform. It was designed to function both to provide a realistic, interactive virtual clinic and instantaneous access to supporting literature on Hunter disease. The *Hunter disease eClinic *consists of an *eBook *and an *eClinic*. The *eClinic *is the interactive virtual clinic component of the software. Within an environment resembling a real clinic, the trainee is instructed to perform a medical history, to examine the patient, and to order appropriate investigation. The program provides clinical data derived from the management of actual patients with Hunter disease. The *eBook *provides instantaneous, electronic access to a vast collection of reference information to provide detailed background clinical and basic science, including relevant biochemistry, physiology, and genetics. In the *eClinic*, the trainee is presented with quizzes designed to provide immediate feedback on both trainee effectiveness and efficiency. User feedback on the merits of the program was collected at several seminars and formal clinical rounds at several medical centres, primarily in Canada. In addition, online usage statistics were documented for a 2-year period. Feedback was consistently positive and confirmed the practical benefit of the program. The online English-language version is accessed daily by users from all over the world; a Japanese translation of the program is also available.

**Conclusions:**

The Hunter disease *eClinic *employs a CBT model providing the trainee with realistic clinical problems, coupled with comprehensive basic and clinical reference information by instantaneous access to an electronic textbook, the *eBook*. The program was rated highly by attendees at national and international presentations. It provides a potential model for use as an educational approach to other rare genetic diseases.

## Background

Learning to manage rare diseases, such as inborn errors of metabolism, presents some serious pedagogical problems. Lysosomal storage diseases present additional challenges to clinicians because they are typically complex disorders, affecting different organ systems and involving multiple medical specialists, including, neurologists, ophthalmologists, cardiologists, rheumatologists, and orthopedic surgeons, as well as general physicians [1]. In the last decade, the emergence of innovative new therapies for these rare diseases [2] has added to the challenge of early diagnosis and appropriate treatment of patients [3].

During their medical undergraduate education and postgraduate specialty training, trainees may not have the opportunity to participate in the care of patients with specific rare genetic disorders [4]. Moreover, the resources needed to manage the complex problems presented by patients are generally not readily available. On the other hand, it is often through detailed studies of the pathophysiology of single-gene disorders, particularly the inborn errors of metabolism, that our understanding of many basic biological principles, applicable throughout medicine, has developed [5].

Computer-based teaching (eLearning) using problem-based teaching holds significant promise for meeting the challenges of medical education [6-12]. Bidwell et al [13] showed that the use of virtual training programs improved student performance in the management of various pediatric problems. Others have generated computer-based case simulations of real-life case scenarios and standardized patient-based assessments which have also proved to be useful tools for enhancing learning[14,15], including the management of surgical problems [16]. Some of these are accessible through web-sites. For example, CAMPUS (http://www.virtual-patients.com) is a web-based teaching system, developed at the University of Heidelberg, Germany, using flexible, case-based training [17]; CLIPP (http://www.clippcases.org) is a multi-institutional, web-based program, which makes use of patient simulations to supplement the core pediatric clerkship curriculum [18].

To our knowledge, ours is the first computer-assisted, problem-based program that specifically addresses learning about the management of a rare, treatable, genetic disease, such as Hunter disease.

### About Hunter disease

Mucopolysaccharidosis type II (Hunter disease) is a rare, progressive, multi-system, lysosomal storage disorder exhibiting a wide range of severity in different patients. It is caused by deficiency of iduronate 2-sulfatase, resulting from mutations in the *IDS *gene. The clinical features of the disease vary markedly among affected patients depending in large part on the level of residual enzyme activity.

The most severe form is characterized by progressive cognitive and neurologic deterioration, airway obstruction and cardiac complications generally culminating in death before age 20. Patients with more attenuated forms of the disease show little or no cognitive impairment, but the involvement of the musculo-skeletal, respiratory and cardiovascular systems is often as debilitating as in patients with severe primary brain involvement, causing cardiac or cardiorespiratory death in early adult years.

The need for general physicians and non-geneticist medical specialists to know more about Hunter disease has become increasingly pressing as a result of the emergence of lysosomal enzyme replacement therapy (ERT), which significantly improves the outcome for patients, but only if the treatment is initiated early, before the development of serious irreversible complications[19-21].

For many years our group, which is part of a research-intensive hospital community, has been involved with the study and management of lysosomal storage diseases (LSDs). A major part of our work has consisted of preparing the next generation of physicians to manage these conditions more confidently through advanced clinical training [22]. The rarity of the disorders underscored for us the need for training resources relying less on actual patient contact, which might only be available in a few highly specialized centers like our own.

Our aim was to develop an *eLearning *tool to provide virtual clinical cases, drawn from real-life situations, with interactive and instantaneous access to a comprehensive multimedia reference book about Hunter disease for use by medical educators, general physicians, pediatricians, and other specialists. We recognized the importance of making the program readily accessible for independent learning since contact with an affected patient, is unpredictable, and what little might have been learned about the disease in medical school or during specialty training would require reinforcement and expansion.

## Implementation and Results

**Figure 1:**
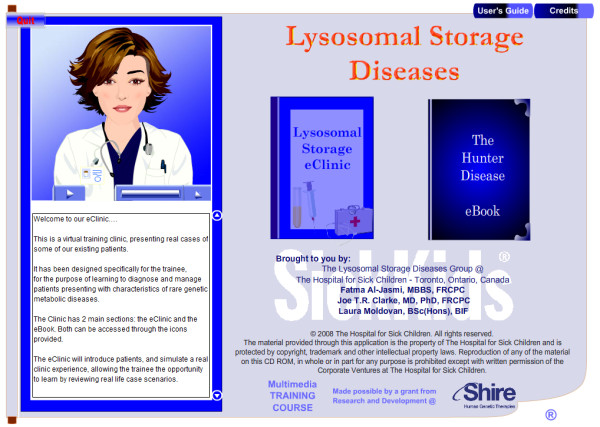
**Screenshot of the *Hunter disease eClinic *entry point showing the "Lysosomal Storage eClinic" and "The Hunter Disease eBook" links**. eBook is the database knowledge about Hunter disease specifics. *eClinic *is the virtual clinic in which the user is guided through disease investigation and management.

We have created an interactive, computer-assisted, problem-based, virtual clinic, the *Hunter disease eClinic*, designed specifically to facilitate independent learning about the management of a rare genetic metabolic disease, Hunter disease, with the needs of a wide range of healthcare professionals in mind. We recognized early the need for two general components to any program designed to meet the needs of independent learners: clinical presentations of virtual patients presenting various clinical problems experienced by patients with Hunter disease (the *eClinic*), and immediate and easy access to a sizable amount of resource information providing background details on the biochemistry, physiology, genetics, complications, and specific treatment of the disease (the *eBook*). We also appreciated the importance of being able to move back and forth easily from one component, the *eClinic*, to the other, the *eBook*, as the learner felt the need or the desire to expand their understanding of specific aspects of the disease (Figure 1).

In the presentation of specific patient problems, we drew on the model of the patient management problem (PMP), a model which employs a highly interactive, stepwise approach to the management of specific patients with multiple opportunities for decision-making, as well as on-going self-evaluation of effectiveness, i.e., getting the right answer, and efficiency, i.e., getting it with a minimum of exploration of irrelevant or extraneous investigation [23]. The final version of the software is accessible free of charge at: http://www.lysosomalstorageresearch.ca

### The eClinic

For the development of the contents of the *eClinic*, we drew heavily, though not exclusively, on cases of MPS II managed at the Hospital for Sick Children and University Health Network in Toronto, collecting clinical details, laboratory data, including the results of biochemical, pedigree, molecular genetic, imaging analyses, as well as digital photos of patients. Formal written parental or patient consent was obtained for the use of any potentially identifying data.

In a typical scenario, the learner is presented with a patient reporting a specific complaint and is asked what additional information he or she would need in order to make a decision about how to deal with the complaint. This invariably involves the need for diagnostic information, and how this is obtained, as well as the results, is a fundamental feature of this approach to learning. Given various options, the learner chooses what amounts to the next step in the problem-solving sequence. The program responds with the desired results, in many cases, the results of a laboratory test. Equipped with the additional information, the learner progresses to the next step and the next decision-making opportunity. Ultimately, the learner reaches a conclusion, which may be a diagnosis, counseling advice, or a specific therapy.

**Figure 2:**
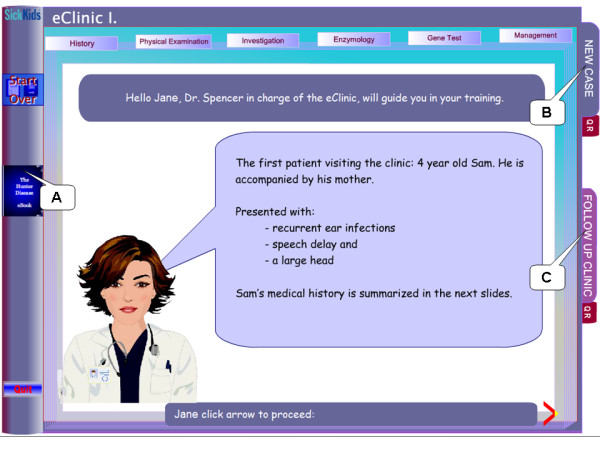
**Screenshot of eClinic training start**. The user has the option of entering his/her name (Jane in this example); the user will be addressed by name throughout the eClinic and recorded in the quiz result. **A**: link to eBook access provided on each page of eClinic. **B**: link access to *eClinic *first module, the guided investigation of a new case. **C**: link access to second *eClinic *module (Follow up clinic). The red directional arrow is the link to linear navigation, to be followed for self-directed training. Each tab and subsequently sub-tab is a link to respective module provided for ease of access and fast reference for later when advanced into the learning experience.

The Hunter *eClinic *consists of two modules, two virtual clinics (see Figure 2), providing an interactive computer simulation of real patients in the context of different clinical scenarios. The first *eClinic *module presents a basic diagnostic problem, focusing on a newly referred patient suspected of having Hunter disease. The second module presents follow-up clinics for known cases of Hunter disease. The follow-up clinic is divided into 7 chapters, describing 7 patients, each presenting with different complaints commonly encountered in affected patients, including a heart murmur, sleep apnea, hand pain, or back pain. It also includes modules covering genetic counseling, risk assessment for Hunter disease, prenatal diagnosis, and treatment by enzyme replacement therapy (ERT).

**Figure 3:**
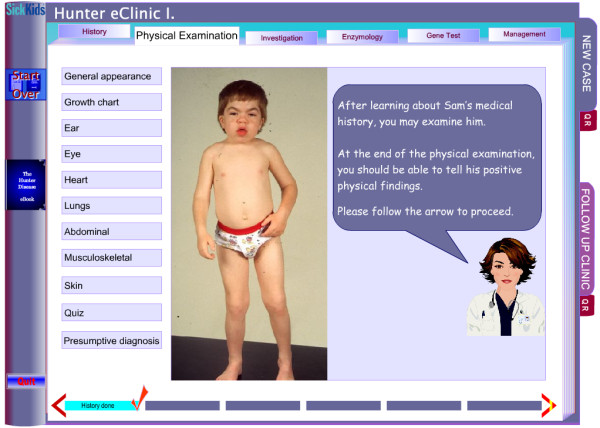
**Screen caption of the *Physical Examination *section in the *eClinic*'s first module**. Note the completed *History *section on the lower line showing user's progress, and the linear course directioning through the red arrows.

The *eClinic *is, therefore, comprised of several clinical scenarios designed to deal with different situations arising in the management of patients with Hunter disease (see Figure 3). Typical situations used for the development of specific scenarios include the initial diagnosis of Hunter disease in a young child with a severe variant of the disease, the identification and management of some specific complications, genetic counseling (including prenatal diagnosis), and ERT.

At various stages throughout each of the *eClinic *modules, the learner is presented with quizzes, providing the opportunity for immediate feedback on performance, as well as easy access to the *eBook*. Typically, the tests are in the form of multiple-choice questions. The learner is given up to three opportunities to answer correctly. On any specific question, the score on effectiveness is based on whether the learner obtained the correct answer or not on the first attempt; the score on efficiency is based on the number of tries the learner takes in order to reach the correct answer.

**Figure 4:**
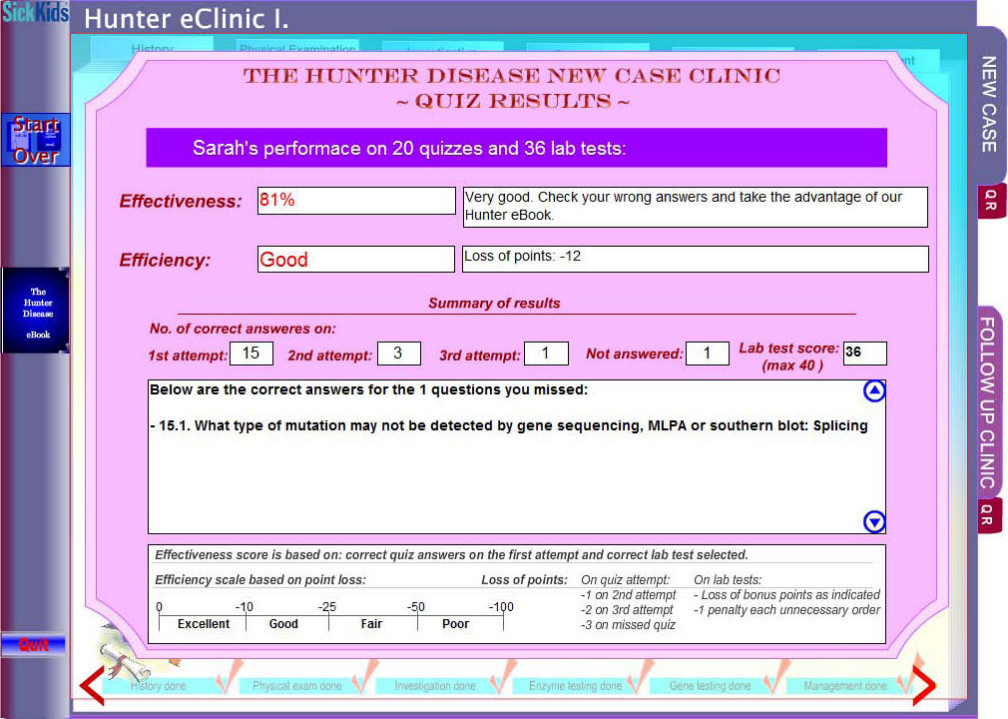
**Screen caption of the results reached upon completion of *eClinic's *first module: New Case**. The performance review reflecting user's progress is given, along with explanations of wrong answers. The page can be printed.

Ultimately, global effectiveness and efficiency scores are calculated on the basis of the cumulative effectiveness scores achieved in the course of answering the short tests that compose each *eClinic *module quiz, and the cumulative efficiency scores, which includes performance on these tests, along with performance on requesting additional information in the course of working up the case (Figure 4).

### The eBook

The *eBook *was developed in response to the need for readily accessible high-quality, resource information to fill in gaps in knowledge. This amounts to an electronic substitute for a textbook, but more easily accessible, comprehensive, relevant and user-friendly.

**Figure 5:**
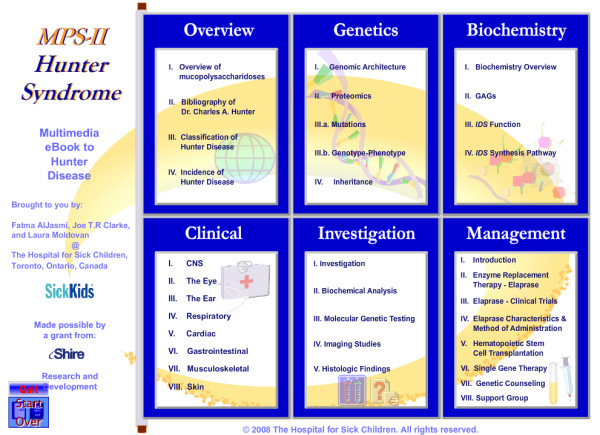
**Screen caption of eBook showing textbook organization of 6 chapters: Overview, Genetics, Biochemistry, Clinical (manifestations), Diagnosis and Management**.

The textbook consists of six chapters: Overview, Genetics, Biochemistry, Clinical Manifestations, Diagnosis and Management (Figure 5). Each chapter contains comprehensive and current knowledge from disciplines contributing to our understanding of Hunter disease and related disorders, presented in a visually engaging manner through graphical animations and hyperlinks to relevant web-sites.

The *Overview *chapter is intended to brief the user on the some historical information, as well as some general characteristics of Hunter disease. The chapter on *Genetics *provides extensive genetic information. Extensive use is made here of hyperlinks to web-sites that are frequently up-dated. In the design of the *Biochemistry *chapter we employed animated graphics to illustrate how iduronate 2-sulfatase is synthesized and processed in a way that would be easily understood by health professionals. The *Clinical *chapter consolidates information derived from a large number of published sources and presents it with generous use of photographs and diagrams, along with references to the published literature. The chapter on *Investigation *provides a roadmap to the diagnostic workup of Hunter disease including clinical biochemistry, molecular genetic testing, imaging studies, and histology. The *Management *chapter includes an extensive treatment of enzyme replacement therapy, as well as coverage of the international Hunter outcome survey and genetic counseling.

One of the key features of the program is the linkage between the *eClinic *and the *eBook*. At virtually any place in the *eClinic*, the learner is able to switch over to the *eBook *in order to check facts or to find out more about some aspect of Hunter disease. The *eBook *is also accessible independently of the *eClinic*, making it a particularly powerful source of a vast amount of information about lysosomal storage diseases in general and about Hunter disease in particular. The material included in the *eBook *is derived primarily from peer-reviewed medical and scientific publications, standard textbooks, trusted electronic databases, and websites maintained by government or institutional agencies. Material or websites supported by pharmaceutical companies, non-profit patient support groups, and other "soft" resources are identified as such. At each stage of its development, the contents of the program were reviewed by a scientific content review committee consisting of a metabolic physician, a biochemist with special expertise in inborn errors of metabolism, a molecular geneticist, and a genetic counselor.

### User feedback

The Hunter *eClinic *Software was presented to pediatric and genetic residents, individually and in groups, at different teaching hospitals in Canada. Formal presentations of the program were also made to potential users at over 20 meetings and symposia, including the 10th International Symposium on MPS and Related Disorders, in Vancouver, in June 2008, where 1200 CD ROMs were distributed (http://www.mpssociety.ca/). In the closing remarks of the conference, the program was described as "the most progressive presentation at the meeting".

A user/audience feedback form was also collected at the meetings and conferences where the program was presented. The feedback was collected in the form of a questionnaire filling form; the questions included rating of the eClinic and eBook chapters, content and information suggestions, general information about the users training background. On average, feedback response rates were ranged between 70 and 75% of the audience. The program consistently received very high or excellent overall scores. The only significant change asked in the feedback forms was a bookmarking and word-search capability, requirements which we were unable to implement owing to the technical limitations of the Flash software.

Although conceived as an educational tool for health professionals, the *Hunter eClinic *has also proven to be a valuable resource for the families of the patients with Hunter disease. As a result of specific interest within the Japanese medical community, the software has been translated into Japanese. An article in the periodical of the Japanese MPS society praised the translation for the help it provided to patients, families, educational and administrative professionals.

Both English and Japanese language versions are accessible online. The number of "hits" per month averages in the range of 12 000 from users from throughout the world; top accessing countries are United States, Canada, Russian Federation, China, Japan, Great Britain, Australia, and the European Union. We were able to collect usage data since January 2010 as the Lysosomal Storage Research Group acquired a unique domain unrestricted by institutional and security related policies.

The Hospital for Sick Children is currently exploring the introduction of a new computerized learning management system (LMS), and *Hunter eClinic *has been selected as one of the backbones in the educational structures; the launching of the system is projected for the fall of 2010.

## Discussion

The Hunter *eClinic *represents an attempt to provide an effective approach to facilitate learning about a rare genetic disease through an interactive program covering the management of specific diagnostic and treatment problems in a virtual clinical setting, coupled with immediate access to a vast amount of resource material, as well as feedback to the learner on their effectiveness and efficiency. The tailoring of the program for independent learning, with the flexibility to respond to a wide range of needs, was considered to be one of the most important design features of the program. Formal exposure to rare diseases, either as medical undergraduates or postgraduate specialty trainees, is unlikely to have been comprehensive or up to date enough to meet the needs of managing affected patients. In the absence of direct exposure, there is little incentive for the learner to invest time in the subject. Faced, then, with a patient affected with a rare disease, the clinician is confronted with the need to learn quickly and efficiently as much as is required to manage the patient's problems confidently. The design features of the program make it particularly well-suited for this kind of situation.

Studies looking at the effectiveness of interactive continuing medical education have shown that individual learning preferences are influenced by factors such as the quality of the program, the degree of self-pacing and self-direction, opportunity for reflection, quantity of interpersonal interaction and educational value of interactions [24]. These factors were carefully taken into consideration in planning the design and contents of the program.

The presentation of the program as two separate, but inter-related, components, the *eClinic *and the *eBook*, combines the strengths of virtual patient management problems with the value of access to the vast amount of published resource information available electronically. The program facilitates constructivist type of knowledge and skill transfer by bringing real world problems into the learning space of *eClinic *and by providing performance feedback. As in real medical clinic, where there is access to textbook and Internet in the *eClinic *the trainee can easily access the *eBook *through an on-screen icon provided throughout the *eClinic*.

The program presents patient scenarios in a linear interactive way. It follows the standard practical path including history taking, physical examination, investigation and management. In order to ensure validity and fidelity the presentation and clinical data of virtual patients were taken from real patients at the Hospital for Sick Children. This ensures that what is learned through *eClinic *is directly transferable to real life scenario. The use of real case scenarios also enhances the engagement of the trainee [25-27].

The *eBook *is enhanced by hyperlinking text to electronically accessible references and websites. This allows the trainees to access additional information easily, providing for more in-depth study. This is a critical step toward self-directed learning [6]. The information provided in the *eBook *is enhanced by the use of Flash animation, taking advantage of the fact that visual perception is the most developed sense in humans. Researchers have shown that animation leads to longer-term memory retention [28]. The program design fulfills the principles of learning as articulated in the practice field theory (Table 1)[29].

**Table 1:** Application of accepted pedagogical principles

***Practice field***		
**Principle 1: doing domain-related practice**	Learners must be actively doing domain-related practice, not listening to the experiences or findings of others.	eClinic provides an opportunity for the trainee to work out the cases.
**Principle 2:ownership of the inquiry**	Learners must see the dilemma as worth investing their efforts. They must feel they are responsible for the solution.	Trainee is addressed by his/her name and gets the sense of responsibility for solving the case. eClinic provides real patient data including the facial features, heart sound, MRI images, skeletal x-ray, blood film etc. It resembles real life clinic environment to ensure engagement.
**Principle 3: coaching and modeling of thinking skills**	The instructor's job (a real instructor or the learning system) is to coach and model learning and problem solving by asking questions that learners should be asking themselves.	Mentor in the eClinic guides the trainee through the clinic and asks questions to stimulate thinking and encourage reading the eBook.
**Principle 4: opportunity for reflection**	Reflection provides individuals with the opportunity to think about why they are doing what they are doing and even to gather evidence to evaluate the efficacy of their decisions. The reflective process is essential to the quality of learning.	The evaluation/feedback system in the eClinc gives the trainee the chance of 3 trials. Where the trainee gets the wrong answer, he will have the chance to rethink or even explore the e-Book for the right answer.
**Principle 5: dilemmas are ill-structured**	Problems to be solved must be either ill-defined or loosely defined only.	eClinic provides a large number of lab tests for the trainee to choose from.
**Principle 6: support the learner rather than simplify the dilemma**	The dilemmas that learners encounter should reflect the complexity of the thinking and work that they are expected to perform in the real world.	eBook provides comprehensive knowledge in one package, combining the basic science and clinical knowledge with links to reference material, journals PubMed citation, publisher of the book, websites etc.
**Principle 7: work is collaborative and social**	The quality and depth of this negotiation and understanding can only be determined in a social environment where ideas are discussed.	Cases in the eClinic can be used by an educator to teach the student and stimulate discussion among the group. Or a group of students can share working out the case and discussing it among themselves. Virtual e-Clinic can be used within a 'real-life" tutorial group with learners working in pairs of threes.
**Principle 8: the learning context is motivating**	Learners must be introduced to the context of problems and their relevance, and this must be done in a way that challenges and engages the learner.	Cases in the eClinic engage the trainee to solve the case. eBook provide lots of animation and interactive tools to stimulate and simplify the complex information. It takes advantages of the visual learning for longer memory retention.

One of the limitations of the *eClinic *program is the lack of an index or an internal search engine enabling the learner to undertake searches for specific topics covered in the *eBook *or the *eClinic*. Instead, the learner must rely on the table of contents to locate a topic of interest and work through the program for the details. By contrast, the hyperlinks embedded in the program provide access to web-based search engines, such as PubMed. A potentially important limitation is the lack of a book-marking capability. Using the CD version of the software, the trainee can stop anywhere in the program and pick up later where she left off; however, once the program is shut off, the results of test performance are lost. In order to take full advantage of the performance feedback features, the learner must complete one of the *eClinic*(s) scenarios in one sitting, or at least without shutting down the program. On the other hand, one of the most appealing features of the *Hunter disease eClinic *is the visual appeal and ease of navigation, features meant to create a positive learning experience.

Owing to the fact that *Hunter eClinic *represents a first of its kind type of educational device, we cannot compare it with other similar projects. Plans for future development versions of the *Hunter disease eClinic *include: incorporating the recent advances in research, analysis and treatment methods and further beta-testing and the incorporation of constructive suggestions for improvements made by experts and non-experts who are viewed as potential users. Feedback collected at future formal presentations of the program at various conferences will be analyzed for ways to optimize it. The possibility is also being explored to enable users to obtain continuing medical education (CME) credits for working through the program.

## Conclusions

The *eClinic *concept provides a useful model for independent learning about rare diseases in general, with specific applicability to lysosomal storage diseases and other inborn errors of metabolism. It provides the user with comprehensive, accurate and up-to-date information, which would normally take a great deal of time and effort to access and acquire through conventional means. While development of the program was both time consuming and costly, over the long-term and with broad use, this system offers significant advantages to learners faced with unusual and challenging clinical situations.

The value of the Hunter disease eClinic as a virtual learning approach in medicine is reflected in feedback from users and particularly in its potential for extending this methodology to other rare genetic diseases.

## Availability and requirements

Project name: HUNTER DISEASE *eCLINIC*

Project distribution: CD ROM and Web - versions

Operating system(s): CD ROM available for Windows and for Apple Macintosh

Project home page: http://www.lysosomalstorageresearch.ca

CD other requirements: The application is available on a CD ROM format, which does not require downloading and installing of additional software on the local computer. The Windows CD ROM is a self-start application, after insertion, the system will read and initiate the applications. The read-response speed will vary to according to the user's computer specifications, 16 sec on average. We have also produced and tested a version that can run on an Apple computer with OS10. On Apple Macintosh Adobe Flash Player version 9 is required, latest Macintosh generations come with all utilities preinstalled.

Disk space available requirement ~ 60 MB: Alternatively, the CD contents may be copied to a computer hard-drive into a separate, newly created folder (the speed of application will increase considerably).

Preferable utilities but not strictly necessary: Audio, Internet Connection, Adobe, Acrobat Reader

**Conditions of use**: The software program material provided through this application was constructed from our comprehensive research on lysosomal storage/Hunter disease and related materials, made possible by an unrestricted educational grant provided by Shire Human Genetic Therapies, and is for educational purposes only. We do not endorse or recommend any commercial products, processes, or services. *Hunter disease eClinic *is the property of The Hospital for Sick Children and is protected by copyright, trademark and other intellectual property laws. The program contains material affecting privacy legislation and copyright restrictions are enforced at all times. Reproduction of any of the material on CD ROM or Web-site in whole or in part for any purpose is prohibited except with written permission of the Corporate Ventures at The Hospital for Sick Children. Otherwise, the contents may be used in whatever way the program meets the personal educational needs of the user.

**Recommended hardware**: for the computer requirements for running the CD ROM see Table 2.

**Table 2:** Computer requirements for running the CD ROM

Windows version	Apple Macintosh version
Microsoft Windows 2000, ME, XP, Vista	Mac OS X v10.1 or later (PowerPC) Mac OS X v10.4 or later (Intel)
2.8 GHz Intel Pentium IV, AMD Athlon and higher 1 Gb of RAM or higher 128 MB Video Card or higher 4X DVD-ROM Drive or higher Resolution 1024 × 768	PowerPC G5 1.8 GHz and higher Intel Core Duo 1.83 GHz and higher 512 MB of RAM (256 MB minimum) 128 MB of VRAM (64 MB minimum) Resolution 1280 × 720 Adobe Flash Player 9
Minimum*: 700(+) MHz Intel Pentium III or AMD Athlon 512 MB of RAM 64 MB Video Card 8X CD-ROM Drive	

*minimum requirements for running the CD ROM: the CD will run however the animations will play at a very low speed or stall considerably. We recommend using the recommended requirements in order to experience the program's capability.

**Web version - tested browsers**: Internet Explorer, Safari, Firefox; the user needs to have macromedia Flash player plug-ins installed, and allow ActiveX controls in order to be able to view it.

**Web version recommendations: The eLearning resources access requires flash**. Make sure ActiveX control is enabled and Flash Player installed. To download the latest Flash player go to: http://get.adobe.com/flashplayer/ and follow the instructions. For reasons of security, institutions may restrict access to Flash Player; check with the local computer tech department if browsing problems are experienced.

To test the computer Flash Player installation: http://kb2.adobe.com/cps/155/tn_15507.html

Flash Player help and support main issues: http://www.adobe.com/support/flashplayer/

For optimal functioning the recommended internet connection is 512 Kbps and higher; 265 Kbps was tested and it work at a slower pace - however once the program is loaded in the browser the navigation is smooth -(within the same open browser window).

The Hunter disease eClinic web version is offered complimentary; if the user's internet connection meets the specifications and browser installed correctly they should be able to access the application; the program was thoroughly tested on various machines, the inability to access the program are related to local machine issues.

Alternatively users may log into the Lysosomal Storage Research community forum which we have provided as a resource for users to ask questions and get help with various related topics.

## List of abbreviations

CAMPUS: Computergestützte Aus- und Weiterbildung in der Medizin mit dem flexiblen und simulativen fallbasierten System (Computer education and training in medicine with the flexible and simulative system, http://www.medicase.de), English website: http://www.virtual-patients.com; CBT: Computer-based teaching; CD ROM: Compact Disk Read Only Memory; CLIPP: Computer-assisted Learning in Pediatrics Program (http://www.clippcases.org); CME: Continuing Medical Education; *e: *electronic; ERT: Enzyme Replacement Therapy; LSDs: Lysosomal Storage Diseases; MPS II: Mucopolysaccharidosis type II also known as Hunter disease; MPSs: Mucopolysaccharidoses; PMP: Patient Management Problem

## Competing interests

The *Hunter disease eClinic *program was developed with the aid of an unrestricted educational grant from Shire Human Genetic Therapies, Inc. The Japanese translation was made possible by an unrestricted educational grant from Genzyme Japan. JTRC has received research and travel grants, as well as honoraria for CME presentations at conferences from Shire HGT, Genzyme Corp, and Actelion Pharmaceuticals.

## Authors' contributions

FAJ wrote the first and subsequent draft versions of the manuscript and the final version of the paper. LM constructed the software, contributed original graphics and figures, assisted with the preparation of the final version of the paper. JTRC developed the concept and overall guidance for the project, as well as contributing to revisions of the manuscript. All authors read and approved the final manuscript.

## Authors' information

FAJ MBBS, FRCPC, former fellow, in The Division of Clinical and Metabolic Genetics at the Hospital for Sick Children in Toronto is a pediatrician and geneticist with special training and practice in lysosomal storage diseases and is a peer-reviewed published author with original articles in medical scientific journals. In 2009 she has accepted an appointment with the Pediatric Department, Faculty of Medicine & Health Sciences, United Arab Emirates University, AlAin, United Arab Emirates

LM BSc(Hons), BIF, is a senior programmer analyst in clinical research and medical computer teaching software in The Division of Clinical and Metabolic Genetics at the Hospital for Sick Children in Toronto with special training in biochemistry, genetics and bioinformatics, and is a peer-reviewed published author in scientific journals of genetics content

JTRC, BSc, MD, MSc (Biochem), PhD (Biochem), MSc (Health Policy), FRCPC, is a Professor of Pediatrics at the University of Toronto and former Director of the Genetic Metabolic Diseases Program at the Hospital for Sick Children in Toronto, as well as Clinical Professor in Medical Genetics at the University of Sherbrooke, Quebec. His research interests include studies on lysosomal storage diseases, especially relating to treatment. Dr. Clarke has authored or co-authored over 200 peer-reviewed original articles in medical scientific journals, in addition to many book chapters and/or books.

## Pre-publication history

The pre-publication history for this paper can be accessed here:

http://www.biomedcentral.com/1472-6920/10/72/prepub
